# Genotype 3 is linked to worse liver disease progression in hepatitis C patients even after SVR following DAA therapy

**DOI:** 10.3389/fcimb.2025.1510939

**Published:** 2025-02-03

**Authors:** Xiping Ran, Yang Xu, Ying Wang, Cheng Zeng, Chen Gong, Ni Wang, Dachuan Cai

**Affiliations:** ^1^ Department of Gastroenterology, Chonggang General Hospital, Chongqing, China; ^2^ Department of Infectious Diseases, The Second Affiliated Hospital, Chongqing Medical University, Chongqing, China; ^3^ Department of Infectious Diseases, Key Laboratory of Molecular Biology for Infectious Diseases (Ministry of Education), Institute for Viral Hepatitis, The Second Affiliated Hospital, Chongqing Medical University, Chongqing, China; ^4^ Department of Neurology, The Second Affiliated Hospital, Chongqing Medical University, Chongqing, China

**Keywords:** hepatitis C virus, direct-acting antiviral, sustained virological response, HCV genotype 3, disease progression

## Abstract

**Background and aims:**

HCV genotype (GT) 3 is associated with rapid liver disease progression. However, the liver disease progression and its risk factors in patients with HCV GT 3 infection after sustained virological response (SVR) following direct-acting antivirals (DAAs) remain unclear. Therefore, we aimed to investigate the liver disease progression of patients with GT 3 after SVR.

**Methods:**

This was a retrospective cohort study of patients with HCV infection who achieved SVR by DAAs. The clinical outcome was overall liver disease progression (OLDP), defined as newly diagnosed compensated liver cirrhosis, decompensated liver cirrhosis, or hepatocellular carcinoma. The incidence of OLDP was evaluated by Kaplan−Meier analysis. Cox regression analysis identified the risk factors for OLDP.

**Results:**

A total of 409 patients (46.9% GT3) were followed for 43.7 (32.9, 58.7) months. The incidence of OLDP was higher in patients with GT 3 (4.63/100PY) than non-GT 3 (0.60/100PY; P < 0.001). According to Cox multivariate analysis, GT 3 was significantly associated with OLDP (HR 6.41, 95% CI 1.82 - 22.56; P=0.004). The predictors of OLDP in patients with GT 3 were HCV recurrence (HR 12.15, 95% CI 3.18 - 46.46; P < 0.001) and FIB-4 > 3.25 (HR 16.40, 95% CI 1.03 - 39.81; P = 0.046) at baseline.

**Conclusion:**

HCV GT 3-infected patients remain at a higher risk of OLDP even after achieving SVR by DAAs, especially patients with advanced liver fibrosis and at high risk for reinfection or virological late relapse.

## Introduction

The World Health Organization estimated that 57.8 million people were living with chronic HCV infection in 2019 and were at risk of subsequent complications, including liver decompensation and hepatocellular cancer (HCC). China currently has the highest number of individuals infected with hepatitis C virus (HCV) in the world (Cui et al., 2023). In previous studies, the most common genotype (GT) of HCV worldwide was genotype (GT) 1 (46%), followed by GT 3 (22%) (Gower et al., 2014). The proportion of GT 3 was 38.7% in southwest China, followed by 13.69% in South China and 11.11% in Northwest China, and the proportion of GT 3 has been increasing each year (Zhou et al., 2009; Chan et al., 2017; Chen et al., 2017; Polaris Observatory HCV Collaborators, 2017; Cui et al., 2023).

Compared with other genotypes, GT 3 is associated with a lower rate of sustained virological response (SVR) and a higher risk of hepatic fibrosis progression and HCC in patients with HCV (Kanwal et al., 2014; Chan et al., 2017; Wu et al., 2020; Wang and Wei, 2021). A study conducted in the United States found that patients infected with GT 3 had a higher risk of end-stage liver disease (ESLD), HCC, and liver-related death compared to those infected with genotype 1. Specifically, these patients had a 1.1-fold increased risk of ESLD, a 2.1-fold increased risk of HCC, and a 1.4-fold increased risk of liver-related death (McMahon et al., 2017). A study conducted in China found that GT 3, particularly subtype 3b, was linked to faster liver disease progression (Wu et al., 2020). However, most conducted on this topic were focused on regions where GT 1 is predominant and the number of GT 3 patients were relatively few in these studies. Therefore, further studies are necessary for regions with high GT 3 prevalence.

In addition, the risk of liver disease progression attributable to HCV infection is decreased by the achievement of sustained virological response (SVR) after direct-acting antivirals (DAAs), which are highly effective in curing more than 90% of patients of all genotypes (Manns et al., 2017; Nagaoki et al., 2020). However, achieving SVR does not equate to complete elimination of the risk of liver disease progression (Semmler et al., 2022). Currently, there is limited data on liver disease progression for GT 3 patients after receiving DAA treatment and achieving SVR.

Therefore, this retrospective cohort study aimed to investigate the influence of GT 3 on liver disease progression for HCV patients who achieved SVR. We hypothesized that GT 3 were associated with worse liver disease progression in HCV patients after SVR following DAA therapy. The findings of this study are significant for formulating appropriate treatment and follow-up strategies for these patients.

## Methods

### Study design and participants

This was a retrospective cohort study of adults (≥18 years) with HCV infection who achieved SVR with DAA treatment at the Second Affiliated Hospital of Chongqing Medical University in China. The patients were treated between August 2015 and September 2021 and followed up through November 2023. The inclusion criteria were as follows: (1) diagnosed with HCV infection; (2) received DAA regimens, which included one of the following: sofosbuvir, sofosbuvir/daclatasvir, sofosbuvir/ledipasvir, sofosbuvir/velpatasvir, elbasvir/grazoprevir, or glecaprevir/pibrentasvir in combination with or without ribavirin (RBV); (3) achieved SVR 12, defined as having undetectable HCV RNA levels for at least 12 weeks after treatment (Yoshida et al., 2015). The exclusion criteria were as follows: (1) failed to achieve SVR; (2) coinfected with hepatitis B virus (HBV) or human immunodeficiency virus (HIV); (3) had a history of HCC and liver transplantation or diagnosed with HCC within 6 months after the end of treatment (EOT); (4) lacked clear genotype results. The choice of the regimens was decided by the physician according to drug policies and clinical practice guidelines at the time of treatment initiation. Patients were followed up every 6 to 12 months after the end of treatment (EOT) until they experienced overall liver disease progression (OLDP) (Kanwal et al., 2014), liver transplantation, or died. This study was approved by the Human Research Ethics Committee.

### Data collection

The following laboratory data at baseline and follow-up visits were collected from the medical records for analysis: anti-HCV, HCV-RNA, HCV genotype, HBV core antibody (anti-HBc), Fibrosis-4 (FIB-4) index; Aspartate aminotransferase to platelet ratio index (APRI) score, white blood cell (WBC), hemoglobin (HB), platelet count (PLT), Albumin (ALB), alanine aminotransferase (ALT), aspartate aminotransferase (AST), alkaline phosphatase (AKP), γ-glutamyl transferase (GGT), alpha-fetoprotein (AFP) and international normalized ratio (INR). The demographic data included age, sex, diabetes status, hypertension status, and alcohol intake. The results of imaging, including abdominal ultrasonography, computed tomography (CT), magnetic resonance (MR), transient elastography (FibroScan^®^, Echosens, Paris, France), and liver pathology, were collected.

### Assessment of variables and outcomes

In this study, the HCV patients were divided into the GT 3 group and the non-GT 3 group. Non-GT 3 included GT1, GT 2, GT 4, and GT 6, except for GT 5 which was not detected. HCV recurrence was defined as patients who achieved SVR 12 but were detected with HCV RNA above the detection limit at least once during the follow-up period. OLDP included newly diagnosed compensated liver cirrhosis (CLC), decompensated liver cirrhosis (DLC), or HCC (Kanwal et al., 2014). In this study, three patients died of gastrointestinal bleeding, one patient died of severe pneumonia, and two patients died because of accidents during FUP. One patient diagnosed with CLC before DAA treatment underwent liver transplantation. Thus, death and liver transplantation were excluded from our analyses of OLDP.

CLC was defined by one of the following criteria: (1) liver biopsy confirming cirrhosis (Metavir or Ishak score); (2) gastroesophageal varices in endoscopy; (3) liver stiffness measurement cutoff >14.6 kPa; (4) imaging studies showing signs of portal hypertension; and (5) laboratory data consistent with 2 out of 4 requirements in the absence of other explanations: PLT <100×10^9/L, serum ALB <35 g/L, INR >1.3, and APRI ≥2 (Tang et al., 2023). DLC was defined as the presence of ascites, variceal bleeding, and/or hepatic encephalopathy on the basis of cirrhosis (Xu et al., 2020). The diagnosis of HCC was based on hepatic histology, AFP, typical CT imaging, or MR imaging findings (Zhou et al., 2023). The diagnosis of fatty liver was based on ultrasound, CT, or MR. Alcohol abuse was defined as the consumption of >50 g/d of alcohol.

### Statistical analysis

All datasets were estimated for normality of variance using the Kolmogorov−Smirnov test (S−K test). Continuous variables are described as the mean (standard deviation) and median (interquartile range [IQR]) according to the normality of the distribution. This study applied the chi-squared (χ²) test or Fisher’s exact test for categorical variables. Student’s t-test (P≥0.05 in the S-K test) or Mann−Whitney U test (P<0.05 in the S-K test) was used for continuous variables according to their normality of distribution to compare baseline characteristics.

The occurrence of OLDP was expressed per 100 patient-years (/100PY) and evaluated based on Kaplan–Meier survival analysis and compared between two groups by the Log-rank test. Significant risk factors linked to OLDP were evaluated through univariate and multivariate Cox regression analyses. The risk factors were expressed as hazard ratios (HR) and 95% confidence intervals (CIs). P<0.05 was considered to indicate statistical significance. Statistical analysis was performed using R version 4.3.0.

## Results

### Patient characteristics

**Figure 1:**
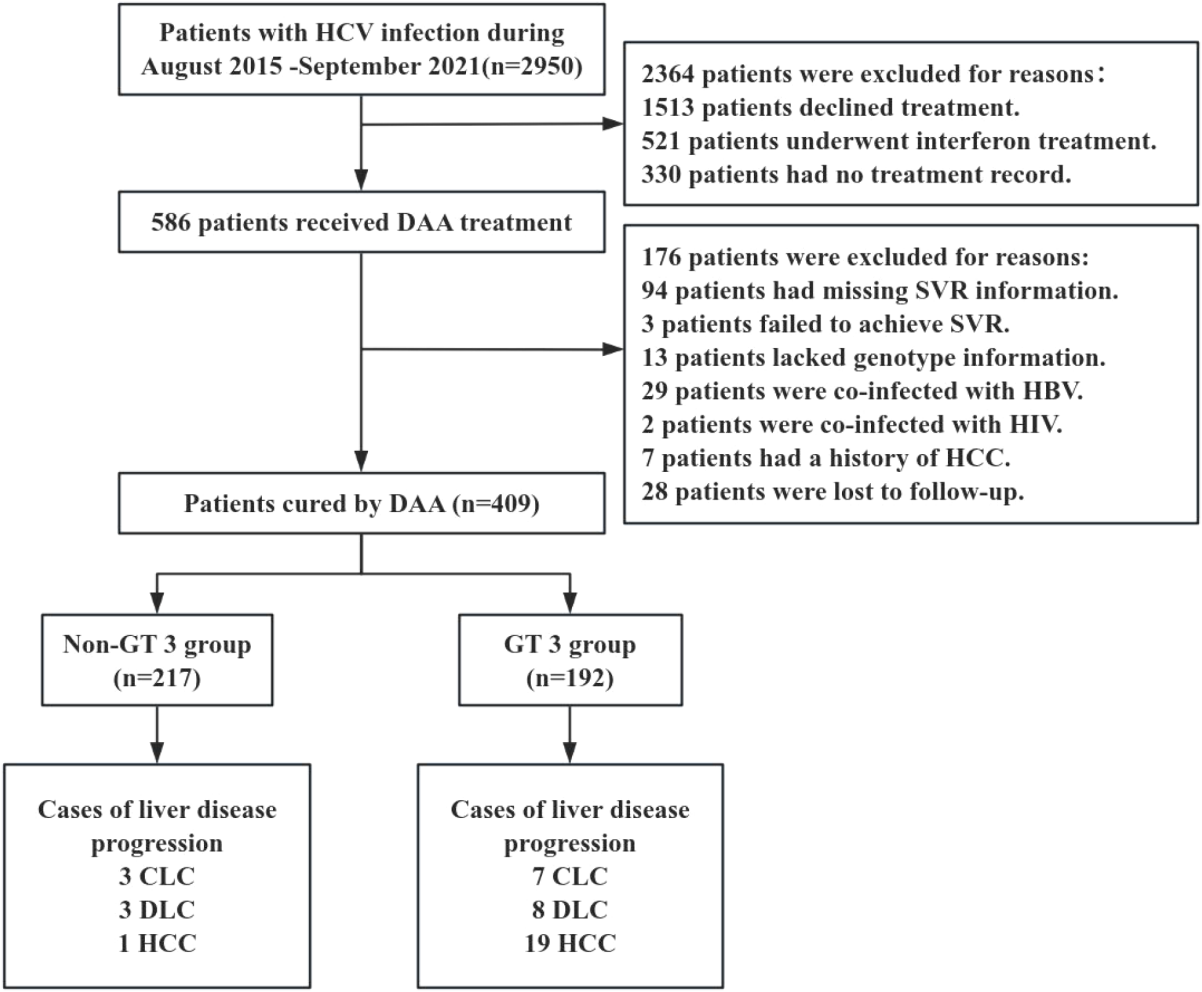
A flowchart of patient selection. HCV, Hepatitis C virus; DAA, Direct-acting antiviral; SVR, Sustained virological response; HBV, Hepatitis B virus; HIV, Human immunodeficiency virus; HCC, Hepatocellular carcinoma; DLC, Decompensated liver cirrhosis; CHC, Chronic hepatitis C; CLC, Compensated liver cirrhosis; GT, Genotype.

From 585 patients who received DAA treatment, 409 patients were included in the current study after strict screening (**Figure 1**). In **Table 1**, 192 (46.9%) patients were divided into the GT 3 group. In the GT 3 group, 94 (49.0%) patients were infected with subtype 3b, and 81 (42.2%) patients were infected with subtype 3a. Additionally, in the non-GT 3 group, the genotypes included GT1 (51/217, 23.5%), GT2 (29/217, 13.4%), GT4 (1/217, 0.5%), and GT6 (136/217, 62.7%) (**Supplementary Table S1**). Compared with the non-GT 3 group, patients with GT 3 were with younger age, lower PLT, higher AFP, higher FIB-4, higher APRI scores, increased risk of baseline cirrhosis and HCV reoccurrence (P<0.05; **Table 1**).

**Table 1:** Baseline characteristics according to the genotypes.

Variables	Total (n = 409)	Non-GT 3 (n = 217)	GT 3 (n = 192)	P
Age, yr.	47 (41, 53)	49 (42, 55)	45 (40, 50)	<0.001
Sex, n(%)				0.697
Female	166 (40.59)	90 (41.47)	76 (39.58)	
Male	243 (59.41)	127 (58.53)	116 (60.42)	
APRI score	1.17 (0.52, 2.37)	0.91 (0.44, 1.90)	1.56 (0.62, 3.27)	<0.001
FIB-4 index	2.49 (1.34, 4.73)	1.85 (1.22, 4.12)	3.09 (1.62, 5.47)	0.002
HCV RNA, log10 IU/mL	6.48 (5.70, 6.96)	6.54 (5.74, 6.97)	6.43 (5.65, 6.95)	0.357
Laboratory study				
WBC, ×10^9/L	5.36 (4.28, 6.58)	5.46 (4.28, 6.58)	5.19 (4.29, 6.63)	0.310
HB, g/L	144 (130, 156)	144 (130, 156)	145 (129, 156)	0.965
PLT, ×10^9/L	151 (101, 201)	169 (121, 206)	130 (84, 188)	<0.001
ALB, g/L	43.5 (40.0, 45.6)	43.8 (40.9, 45.7)	42.7 (38.7, 45.4)	0.045
ALT, IU/L	72.5 (39.0, 126.0)	64.0 (37.0, 123.8)	76.5 (41.3, 129.8)	0.472
AST, IU/L	60.5 (36.3, 95.8)	54.5 (34.0, 82.3)	64.5 (40.3, 109.0)	0.031
AKP, IU/L	81.0 (66.0, 104.0)	84.5 (68.8, 101.0)	78.0 (61.0, 108.0)	0.210
GGT, IU/L	56.0 (28.0, 99.5)	52.0 (26.0, 91.5)	66.5 (31.8, 115.0)	0.061
TBIL, μmol/L	12.9 (9.3, 17.8)	12.4 (9.4, 17.0)	14.0 (9.3, 18.5)	0.258
AFP, ng/dL	7.97 (4.41, 13.88)	5.86 (4.07, 11.19)	8.77 (5.28, 14.92)	0.021
Combination with RBV, n(%)				<0.001
No	188 (45.97)	138 (63.59)	50 (26.04)	
Yes	221 (54.03)	79 (36.41)	142 (73.96)	
Diabetes, n(%)				0.737
No	381 (93.15)	203 (93.55)	178 (92.71)	
Yes	28 (6.85)	14 (6.45)	14 (7.29)	
Hypertension, n(%)				0.530
No	371 (90.71)	195 (89.86)	176 (91.67)	
Yes	38 (9.29)	22 (10.14)	16 (8.33)	
Alcohol abuse, n(%)				0.225
No	352 (86.06)	191 (88.02)	161 (83.85)	
Yes	57 (13.94)	26 (11.98)	31 (16.15)	
Anti-HBc, n(%)				0.558
Negative	268 (65.53)	145 (66.82)	123 (64.06)	
Positive	141 (34.47)	72 (33.18)	69 (35.94)	
Disease status, n(%)				0.002
CHC	262 (64.06)	153 (70.51)	109 (56.77)	
CLC	118 (28.85)	56 (25.81)	62 (32.29)	
DLC	29 (7.09)	8 (3.69)	21 (10.94)	
Fatty liver, n(%)				0.041
No	378 (92.42)	206 (94.93)	172 (89.58)	
Yes	31 (7.58)	11 (5.07)	20 (10.42)	
HCV recurrence, n(%)				0.389
No	400 (97.80)	214 (98.62)	186 (96.88)	
Yes	9 (2.20)	3 (1.38)	6 (3.12)	
Follow-up duration, mo.	40.6 (32.2, 57.8)	46.7 (34.7, 59.8)	38.9 (27.0, 54.9)	<0.001

GT, Genotype; RBV, Ribavirin; CHC, Chronic hepatitis C; CLC, Compensated liver cirrhosis; DLC, Decompensated liver cirrhosis; Anti-HBc, Hepatitis B core antibody; FIB-4, Fibrosis-4; APRI, Aspartate aminotransferase to platelet ratio index; HCV, Hepatitis C virus; WBC, White blood cell; HB, Hemoglobin; PLT, Platelet count; ALB, Albumin; ALT, Alanine aminotransferase; AST, Aspartate aminotransferase; AKP, Alkaline phosphatase; GGT, γ-glutamyl transferase; TBIL, Total bilirubin; AFP, Alpha-fetoprotein; SVR, Sustained virological response; yr., Year; Mo., Month.

### Incidences of overall liver disease progression

The median time from EOT to OLDP during follow-up in patients with GT 3 was shorter than that in patients with non-GT 3 (38.9 vs. 46.7 months; P<0.001; **Table 1**). Overall, OLDP occurred in 10 patients with CLC, 11 patients with DLC, and 20 patients with HCC. In the GT 3 group, the incidences of newly diagnosed CLC, DLC, and HCC were 3.65% (7/219), 4.17% (8/219), and 9.90% (19/219), respectively. The non-GT 3 group had lower incidences of 1.38% (3/217) for CLC and DLC, and 0.46% (1/217) for HCC. Regarding the median progression time, the GT 3 group had intervals of 11.47 months (CLC), 7.42 months (DLC), and 18.70 months (HCC), while the non-GT 3 group had 34.17 months (CLC), 25.53 months (DLC), and 48.30 months (HCC). The incidence of OLDP was higher in patients with GT 3 (4.63/100PY, 95% CI 3.24-6.57) than non-GT 3 (0.60/100PY, 95% CI 0.26-1.48; P <0.001; **Table 2**). The populations with high incidence of OLDP (>5.00/PY) were GT 3-infected with male, cirrhosis, diabetes, anti-HBC positive, FIB-4>3.25, WBC<4×10^9/L, PLT<100×10^9/L, ALB<40 g/L, TBIL>28μmol/L and HCV recurrence (**Table 2**).

**Table 2:** Incidence of OLDP in patients with HCV GT 3 after SVR according to different subgroups.

Subgroups	GT 3			Non-GT 3			P
	PY	Number of patients	Incidence 100PY (95% CI)	PY	Number of patients	Incidence 100PY (95% CI)	
All enrolled patients	34	192	4.63 (3.24-6.57)	7	217	0.60 (0.26-1.40)	<0.001
Sex							
Female	259	6	2.32 (1.07-4.97)	349	2	0.57 (0.16-2.06)	0.083
Male	367	23	6.27 (4.21-9.23)	482	3	0.62 (0.21-1.81)	<0.001
Combination with RBV							
No	156	7	4.49 (2.19-8.98)	493	3	0.61 (0.21-1.78)	0.001
Yes	470	22	4.68 (3.11-6.98)	337	2	0.59 (0.16-2.13)	0.001
Cirrhosis							
No	351	9	2.56 (1.35-4.80)	594	3	0.51 (0.17-1.48)	0.009
Yes	275	20	7.27 (4.75-10.96)	236	2	0.85 (0.23-3.04)	0.001
Diabetes							
No	578	23	3.98 (2.67-5.90)	781	4	0.51 (0.20-1.31)	<0.001
Yes	48	6	12.50 (5.86-24.70)	49	1	2.04 (0.36-10.69)	0.064
Anti-HBc							
Negative	410	14	3.41 (2.04-5.64)	566	1	0.18 (0.03-1.00)	<0.001
Positive	216	15	6.94 (4.25-11.13)	264	4	1.52 (0.59-3.84)	0.004
FIB-4							
≤3.25	207	2	0.97 (0.27-3.46)	317	2	0.63 (0.17-2.27)	0.602
>3.25	294	22	7.48 (4.99-11.07)	309	2	0.65 (0.18-2.33)	<0.001
WBC, ×10^9/L							
<4	108	13	12.04 (7.17-19.51)	113	1	0.88 (0.15-4.83)	0.001
≥4	412	12	2.91 (1.67-5.02)	554	3	0.54 (0.18-1.58)	0.003
PLT, ×10^9/L							
<100	249	19	7.63 (4.94-11.61)	466	2	0.43 (0.12-1.55)	0.027
≥100	270	6	2.22 (1.02-4.76)	204	2	0.98 (0.27-3.50)	0.002
ALB, g/L							
<40	153	14	9.15 (5.53-14.77)	501	2	0.40 (0.11-1.44)	0.003
≥40	363	10	2.75 (1.50-4.99)	133	1	0.75 (0.13-4.13)	0.003
AKP, IU/L							
≤135	439	20	4.56 (2.97-6.94)	573	3	0.52 (0.18-1.52)	<0.001
>135	50	1	2.00 (0.35-10.50)	48	1	2.08 (0.37-10.89)	0.982
TBIL, μmol/L							
≤28	470	19	4.04 (2.60-6.22)	614	4	0.65 (0.25-1.66)	<0.001
>28	36	5	13.89 (6.08-28.66)	18	0	0	0.207
HCV Recurrence							
No	614	24	3.91 (2.64-5.75)	820	5	0.61 (0.26-1.42)	<0.001
Yes	12	5	41.67 (19.33-68.05)	10	0	0	0.055

OLDP, Overall liver disease progression; SVR, Sustained virological response; PY, Person-year; GT, Genotype; RBV, Ribavirin; Anti-HBc, Hepatitis B core antibody; FIB-4, Fibrosis-4; HCV, Hepatitis C virus; PLT, Platelet count; ALB, Albumin; AKP, Alkaline phosphatase; TBIL, Total bilirubin.

**Figure 2:**
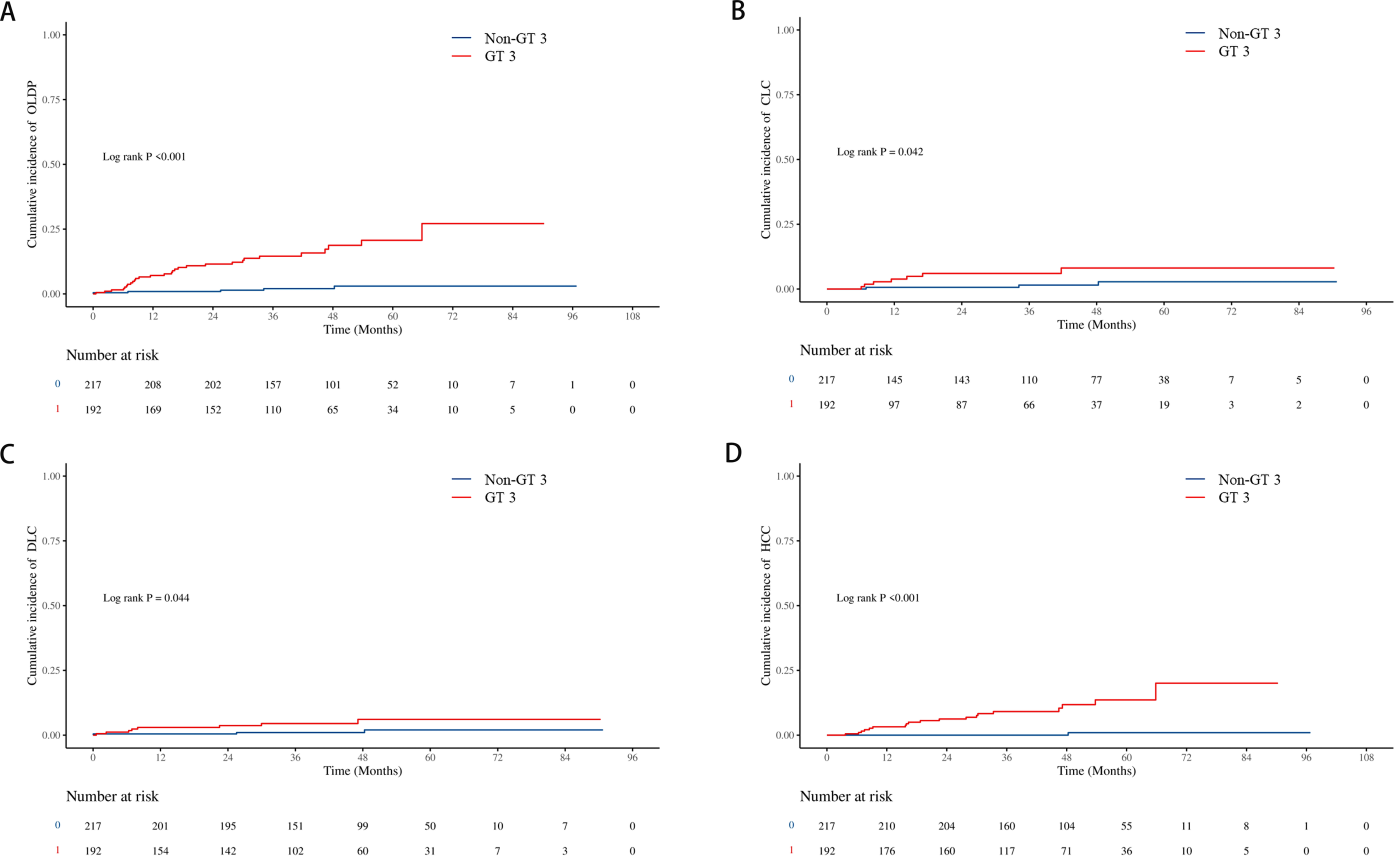
Cumulative incidence of liver disease progression in the GT 3 group and non-GT 3 group. **(A)** The cumulative incidence rates of OLDP; **(B)** The cumulative incidence rates of CLC; **(C)** The cumulative incidence rates of DLC; **(D)** The cumulative incidence rates of HCC. The level of significance was set at p<0.05 (Kaplan−Meier estimates). GT, genotype; OLDP, overall liver disease progression; CLC, Compensated liver cirrhosis; DLC, Decompensated liver cirrhosis; HCC, hepatocellular carcinoma.

**Figure 3:**
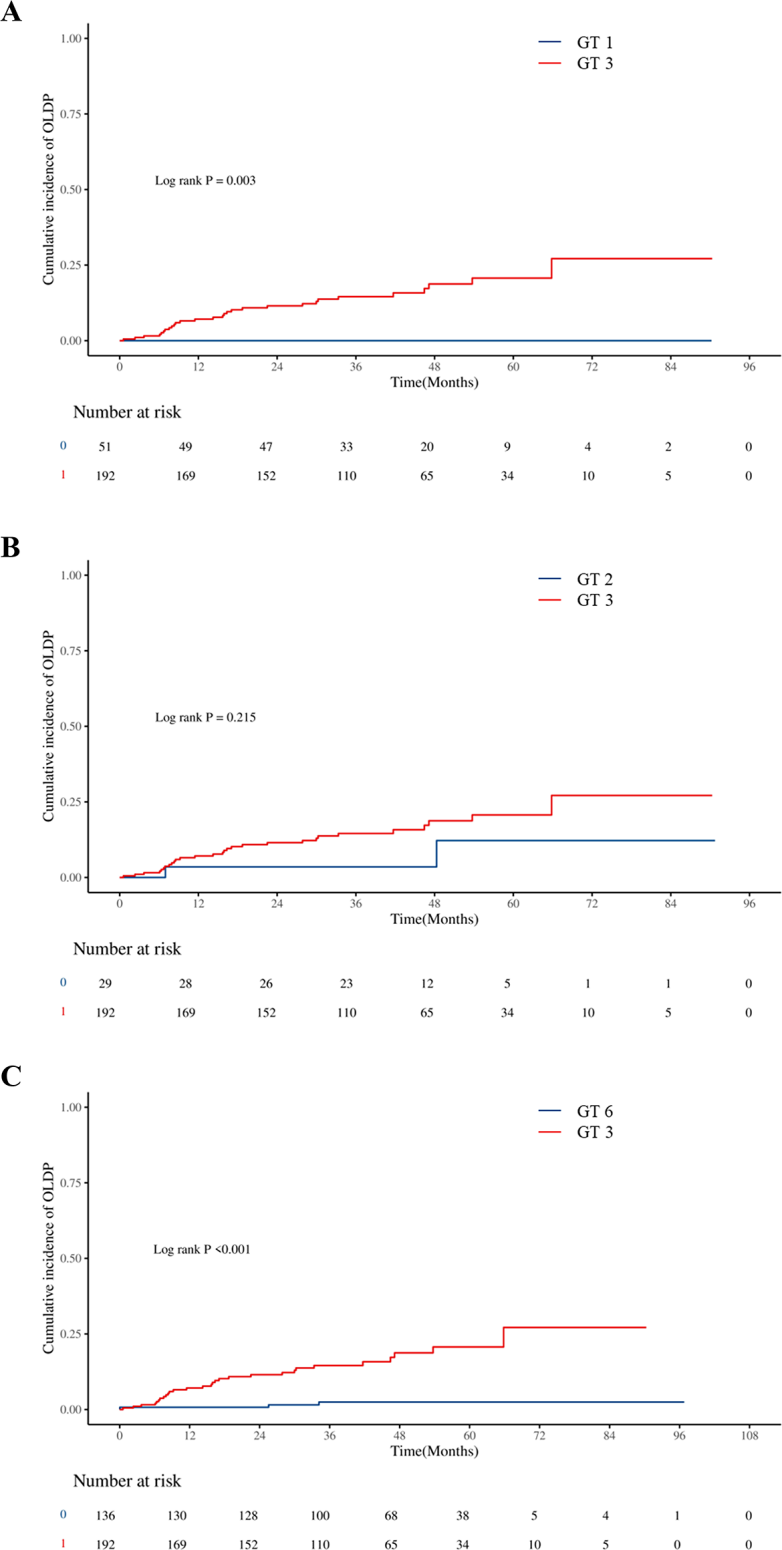
Cumulative incidences of liver disease progression in patients: **(A)** between GT 3 and GT 1, **(B)** between GT 3 and GT 2, and **(C)** between GT 3 and GT 6.

**Figure 4:**
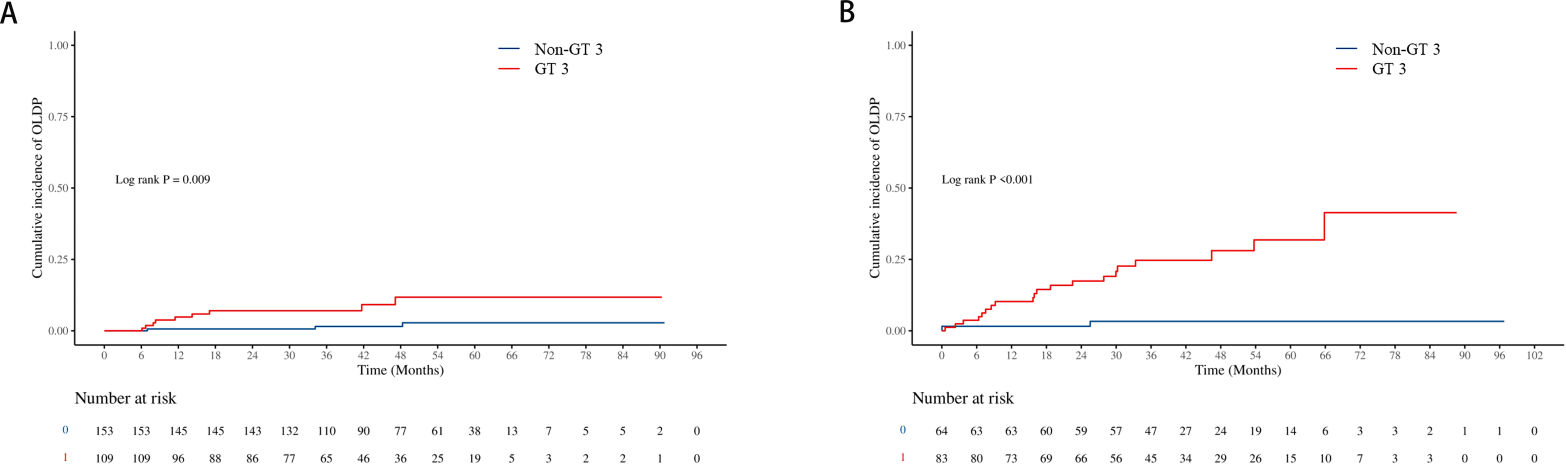
Cumulative incidence of OLDP according to cirrhosis status. The cumulative incidence rates of OLDP in the patients **(A)** without cirrhosis and **(B)** with cirrhosis. The level of significance was set at p<0.05 (Kaplan−Meier estimates). GT, genotype; OLDP, overall liver disease progression.

The cumulative incidence of OLDP at 5 years was higher for patients with GT 3 (18.7%) than for those with non-GT 3 (3.0%; P<0.001), as was the case with CLC (7.8% vs. 2.8%, P=0.042), DLC (5.9% vs. 2.0%, P = 0.044), and HCC (12.7% vs. 1.0%, P < 0.001) (**Figure 2**). The cumulative incidence of OLDP in patients with GT 3 was higher than that with GT 1 (P=0.003) and GT 6 (P<0.001) (**Figure 3**), but there was no statistical difference from that in patients with GT 2 (P=0.215). Among 262 patients without cirrhosis, those infected with GT 3 had a higher cumulative incidence of OLDP at 5 years than those infected with non-GT 3 (11.1% vs. 2.8%; P=0.009) (**Figure 4A**). Patients with cirrhosis in the GT 3 group had a higher risk of OLDP than those with non-GT 3 (27.3% vs. 3.3%; P<0.001) (**Figure 4B**). After excluding the patients with HCV recurrence, the cumulative incidence of OLDP in GT 3 patients was statistically different from that in non-GT 3 patients (P < 0.001). There was no statistical difference in the cumulative incidence of CLC (P=0.172) and DLC (P=0.081) between the two groups. The cumulative incidence of HCC after completion of DAA treatment and SVR was statistically different between the two groups (P < 0.001).

### Risk factors of overall liver disease progression in all patients

In **Table 3**, univariate analysis results suggested that male, diabetes, HCV recurrence, FIB-4>3.25, PLT<100×10^9/L, and ALB<40 g/L at baseline were associated with OLDP risk. Furthermore, according to multivariate Cox regression analysis, the predictors of OLDP in all patients were male (HR 3.23, 95% CI 1.10 - 9.50; P=0.033), GT 3 (HR 6.41, 95% CI 1.82 – 22.56; P=0.004) and HCV recurrence (HR 7.90, 95% CI 2.35 – 26.58; P<0.001). In **Supplementary Table S2**, Cox regression analysis showed that the FIB-4 index (HR 6.40, 95% CI 1.03 – 39.81) and HCV recurrence (HR 12.15, 95% CI 3.18 – 46.46) are risk factors of OLDP for GT 3 patients.

**Table 3:** Variables associated with OLDP in patients with all patients (univariate and multivariate analysis).

	Univariate and multivariate analysis			Multivariate analysis		
	Z	*P*	HR (95%CI)	Z	*P*	HR (95%CI)
Cirrhosis						
No			1.00 (Reference)			1.00 (Reference)
Yes	3.39	<0.001	3.38 (1.67 ~ 6.83)	-0.67	0.502	0.61 (0.15 ~ 2.55)
Gender						
Female			1.00 (Reference)			1.00 (Reference)
Male	2.05	0.041	2.29 (1.04 ~ 5.05)	2.13	0.033	3.23 (1.10 ~ 9.50)
Genotype						
Non-GT3			1.00 (Reference)			1.00 (Reference)
GT3	4.16	<0.001	7.50 (2.90 ~ 19.38)	2.89	0.004	6.41 (1.82 ~ 22.56)
Combination with RBV						
No			1.00 (Reference)			
Yes	1.83	0.068	1.99 (0.95 ~ 4.16)			
Diabetes						
No			1.00 (Reference)			1.00 (Reference)
Yes	2.98	0.003	3.55 (1.54 ~ 8.15)	1.51	0.132	2.04 (0.81 ~ 5.15)
Fatty Liver at baseline						
No			1.00 (Reference)			
Yes	-0.88	0.376	0.41 (0.06 ~ 2.98)			
Fatty Liver during FUP						
No			1.00 (Reference)			
Yes	-1.27	0.204	0.28 (0.04 ~ 2.01)			
Anti-HBc						
Negative			1.00 (Reference)			1.00 (Reference)
Positive	2.68	0.007	2.53 (1.28 ~ 4.98)	0.97	0.334	1.51 (0.65 ~ 3.51)
HCV Recurrence						
No			1.00 (Reference)			1.00 (Reference)
Yes	4.70	<0.001	9.97 (3.82 ~ 25.99)	3.34	<0.001	7.90 (2.35 ~ 26.58)
FIB-4 Index						
≤3.25			1.00 (Reference)			1.00 (Reference)
>3.25	3.07	0.002	5.25 (1.82 ~ 15.14)	1.65	0.100	3.52 (0.79 ~ 15.77)
WBC, 10^9/L						
≥4			1.00 (Reference)			1.00 (Reference)
<4	3.78	<0.001	4.08 (1.97 ~ 8.47)	1.23	0.217	1.78 (0.71 ~ 4.45)
PLT, 10^9/L						
≥100			1.00 (Reference)			1.00 (Reference)
<100	3.51	<0.001	4.32 (1.91 ~ 9.77)	0.78	0.436	1.61 (0.48 ~ 5.37)
ALB, g/L						
≥40			1.00 (Reference)			1.00 (Reference)
<40	3.43	<0.001	3.79 (1.77 ~ 8.10)	1.10	0.273	1.71 (0.65 ~ 4.47)
AKP, IU/L						
<135			1.00 (Reference)			
≥135	-0.20	0.844	0.87 (0.20 ~ 3.67)			
TBIL, μmol/L						
<28			1.00 (Reference)			1.00 (Reference)
≥28	2.98	0.003	4.35 (1.65 ~ 11.45)	0.12	0.905	1.07 (0.36 ~ 3.17)

OLDP, Overall liver disease progression; SVR, Sustained virological response; PY, Person-year; HCV, Hepatitis C virus; GT, Genotype; RBV, Ribavirin; Anti-HBc, Hepatitis B core antibody; FIB-4, Fibrosis-4; HCV, Hepatitis C virus; PLT, Platelet count; ALB, Albumin; HR, Hazard ratio; CI, Confidence interval.

## Discussion

The risk of liver disease progression was decreased after viral eradication, while this risk could not be eliminated in some patients with certain characteristics. To our knowledge, this is the first study to investigate the clinical outcomes of patients infected with GT 3 who achieved DAA-induced SVR. Our findings revealed that patients with GT 3 were at a higher risk of OLDP including HCC after successful DAA therapy than those with non-GT 3. In addition, we reported the post-SVR incidences of OLDP in each subgroup among patients with GT 3 and non-GT 3, which has never been reported before. Finally, we identified the risk factors of OLDP in patients with GT 3 after DAA therapy and built a model to predict this risk for clinical practice.

In our study, 46.9% of patients treated with DAAs were infected with GT 3. This proportion was higher than that in most previous studies, which reported that the proportion of patients with GT 3 was 8.3-13.8% (Ioannou et al., 2017; Nahon et al., 2018). In China, HCV GT 3 (33.1-39.4%) was more prevalent in southwest provinces, such as Yunnan, Guangxi, and Chongqing (Zhou et al., 2009; Chen et al., 2017; Tang et al., 2023). Southwestern China is a vital transit area for drug smuggling, and intravenous drug abuse has become the major risk factor for GT 3 (Ruta and Cernescu, 2015; Chen et al., 2017; Wu et al., 2020). This may explain the high prevalence of GT 3 in southwestern China. In this study, we found that the GT 3 group was younger (45 vs 49) and had a higher proportion of cirrhosis (43.2% vs 29.5%) when treated with DAAs. Patients with GT 3 had a shorter median time (38.9 vs 46.7) from EOT to experience their first event of OLDP. Similarly, a Chinese study reported a younger age and shorter duration from infection to liver disease progression in GT 3-infected patients than in patients with other genotypes (Lu et al., 2017; Wu et al., 2020).

In terms of clinical outcomes, several cohort studies have reported GT 3 is related to a higher rate of liver disease progression, including cirrhosis and HCC, and liver-related death compared to other genotypes (Kanwal et al., 2014; McMahon et al., 2017; Lee et al., 2019). These findings are similar to our study, a higher incidence of liver disease progression was observed in patients with GT 3 in this study. The same conclusion was obtained in the subgroup analysis. Biopsy studies showed that GT 3 was demonstrated to be much more likely to increase liver steatosis than other genotypes, resulting in more rapid liver fibrosis which leads to higher incidence rates of cirrhosis and HCC (Abid et al., 2005; Probst et al., 2011). Previous studies revealed an improvement in liver steatosis in patients with GT 3 after antiviral treatment but not in patients with GT 1, which seems to indirectly explain this association (Zarębska-Michaluk, 2021). However, whether GT 3-induced liver steatosis persists after virus eradication remains unclear. We have observed that GT 3-infected patients without cirrhosis are more likely to develop new cirrhosis, while we found that fatty liver mainly diagnosed by ultrasound at baseline is not associated with liver disease progression. The reason is probably a missed diagnosis of fatty liver by ultrasound. The lack of data, including body mass index and controlled attenuation parameter, hinders further analysis of the association of GT 3 and liver steatosis after DAA therapy. Relevant studies based on DAAs are needed in the future.

Our results displayed that FIB-4 index and HCV recurrence were risk factors of OLDP for GT 3 patients. Few studies reported the effects of reinfection and late HCV recurrence on disease progression after DAA treatment. This study suggested that HCV recurrence, regardless of the cause, increased the risk of liver disease progression after SVR following DAA. High-risk behaviors (such as MSM, drug injection, etc.) that can lead to reinfection should be avoided after successful antiviral treatment. In this study, FIB-4 was used to evaluate liver fibrosis to explore the impact of liver fibrosis on the risk of liver disease progression. Among patients with FIB-4>3.25 and APRI<2, 3/58 (5.2%, 1.29/100 PY) developed HCC, and the incidence of HCC was similar to that in the study of Alessia (6/133, 1.22/100 PY) (Ciancio et al., 2023). In this study, there were 170 patients with FIB-4 > 3.25, of whom 29 were not diagnosed with cirrhosis, and 2/29 progressed to cirrhosis after antiviral treatment, which suggested that not all patients with liver fibrosis could achieve fibrosis regression after DAA treatment. Dynamic follow-up of FIB-4 and imaging is necessary. Similarly, Ioannou observed that FIB-4 continued to rise after SVR (Ioannou et al., 2019). However, unrecognized cirrhosis may be present in non-cirrhotic patients with high FIB-4, while patients diagnosed with cirrhosis and with low FIB-4 may not actually have cirrhosis. FIB-4, as a classic noninvasive diagnostic indicator for fibrosis, still has some limitations, and future studies need to combine more indicators to stratify the risk of liver disease progression after DAA therapy.

Several limitations should be noted when interpreting our findings. First, this was a retrospective study. Patients were enrolled from a single center in southwestern China, which may limit the generalizability of our results to other populations. Second, the diagnosis of cirrhosis and HCC is considerably dependent on imaging methods, which could miss the diagnosis of very early cirrhosis and HCC. Third, this study did not calculate the mortality of such a cohort, which was mainly affected by non-hepatic causes. Published research has demonstrated that DAA therapy is independently associated with a significant decrease in the risk of mortality, which may lead to a lower incidence of liver disease progression. Fourth, due to the natural of retrospective study design, the lack of BMI, LSM values, CAP scores, alcohol consumption after SVR were not analyzed in this study, which may provide more information on the liver disease progression after SVR. Finally, the patients lost to follow-up could potentially introduce bias.

## Conclusion

This retrospective cohort study proved the higher risk of liver disease progression of GT 3-infected patients even if achieving SVR by successful DAA therapy. It reinforced the need for the surveillance of GT 3-infected patients after treatment, especially with FIB-4>3.25 and at the risk of HCV recurrence.

## Data Availability

The raw data supporting the conclusions of this article will be made available by the authors, without undue reservation.
