# Supplementary material for: Genotype 3 is linked to worse liver disease progression in hepatitis C patients even after SVR following DAA therapy
Source: Front Cell Infect Microbiol. 2025 Feb 3;15:1510939. doi: 10.3389/fcimb.2025.1510939 (PMC11830654; doi:10.3389/fcimb.2025.1510939)

**Supplemental Material: Genotype 3 is linked to worse liver disease progression in hepatitis C patients** **even after SVR following DAA therapy**

**Table S1 Genotype distribution of screening participants**

| Genotype | Number of patients |
| --- | --- |
| 1 | 51 (12.5%) |
| 2 | 29 (7.1%) |
| 3  3a  3b  3k  3g  3d  3e/d  Undefined subtype | 192 (46.9%)  80  94  14  1  1  1  1 |
| 4 | 1 (0.2%) |
| 6 | 136 (33.3%) |

**Table S2 Variables associated with OLDP in patients with HCV GT 3 (univariate and** **multivariate analysis)**

|  | Univariate and multivariate analysis | | |  | Multivariate analysis | | |
| --- | --- | --- | --- | --- | --- | --- | --- |
|  | Z | *P* | HR (95%CI) |  | Z | *P* | HR (95%CI) |
| Age |  |  |  |  |  |  |  |
| ≥45 |  |  | 1.00 (Reference) |  |  |  | 1.00 (Reference) |
| ＜45 | 0.61 | 0.539 | 1.26 (0.60 ~ 2.64) |  | -0.33 | 0.745 | 0.84 (0.31 ~ 2.34) |
| Sex |  |  |  |  |  |  |  |
| Female |  |  | 1.00 (Reference) |  |  |  | 1.00 (Reference) |
| Male | 2.13 | 0.033 | 2.66 (1.08 ~ 6.54) |  | 1.69 | 0.092 | 2.73 (0.85 ~ 8.75) |
| Subtypes of GT 3 |  |  |  |  |  |  |  |
| 3a |  |  | 1.00 (Reference) |  |  |  | 1.00 (Reference) |
| 3b | -0.36 | 0.720 | 0.87 (0.41 ~ 1.86) |  | -0.37 | 0.710 | 0.84 (0.34 ~ 2.08) |
| Others | -0.62 | 0.534 | 0.62 (0.14 ~ 2.76) |  | 0.80 | 0.424 | 1.92 (0.39 ~ 9.54) |
| Combination with RBV |  |  |  |  |  |  |  |
| No |  |  | 1.00 (Reference) |  |  |  | 1.00 (Reference) |
| Yes | 0.14 | 0.888 | 1.06 (0.45 ~ 2.49) |  | 0.27 | 0.789 | 1.20 (0.31 ~ 4.63) |
| Diabetes |  |  |  |  |  |  |  |
| No |  |  | 1.00 (Reference) |  |  |  | 1.00 (Reference) |
| Yes | 2.45 | 0.014 | 3.08 (1.25 ~ 7.58) |  | 1.58 | 0.114 | 2.60 (0.80 ~ 8.53) |
| Alcohol abuse |  |  |  |  |  |  |  |
| No |  |  | 1.00 (Reference) |  |  |  | 1.00 (Reference) |
| Yes | 1.53 | 0.127 | 1.89 (0.83 ~ 4.28) |  | -0.87 | 0.384 | 0.63 (0.22 ~ 1.80) |
| Anti-HBc |  |  |  |  |  |  |  |
| Negative |  |  | 1.00 (Reference) |  |  |  | 1.00 (Reference) |
| Positive | 1.82 | 0.069 | 1.97 (0.95 ~ 4.08) |  | 0.44 | 0.659 | 1.21 (0.51 ~ 2.88) |
| HCV Recurrence |  |  |  |  |  |  |  |
| No |  |  | 1.00 (Reference) |  |  |  | 1.00 (Reference) |
| Yes | 4.39 | <.001 | 8.97 (3.37 ~ 23.87) |  | 3.65 | <.001 | 12.15 (3.18 ~ 46.46) |
| FIB-4 index |  |  |  |  |  |  |  |
| ≤3.25 |  |  | 1.00 (Reference) |  |  |  | 1.00 (Reference) |
| ＞3.25 | 2.75 | 0.006 | 7.65 (1.80 ~ 32.58) |  | 1.99 | 0.046 | 6.40 (1.03 ~ 39.81) |
| PLT, 10^9/L |  |  |  |  |  |  |  |
| ≥100 |  |  | 1.00 (Reference) |  |  |  | 1.00 (Reference) |
| ＜100 | 2.64 | 0.008 | 3.46 (1.38 ~ 8.69) |  | 0.52 | 0.601 | 1.39 (0.40 ~ 4.86) |
| ALB, g/L |  |  |  |  |  |  |  |
| ≥40 |  |  | 1.00 (Reference) |  |  |  | 1.00 (Reference) |
| ＜40 | 2.82 | 0.005 | 3.22 (1.43 ~ 7.26) |  | 1.45 | 0.148 | 1.94 (0.79 ~ 4.76) |

OLDP: Overall liver disease progression; SVR: Sustained virological response; PY: Person-year; HCV: Hepatitis C virus; GT: Genotype; RBV: Ribavirin; Anti-HBc: Hepatitis B core antibody; FIB-4: Fibrosis-4; HCV: Hepatitis C virus; PLT: Platelet count; ALB: Albumin; HR: Hazard ratio; CI: Confidence interval.

**Figure S1** Cumulative incidence of liver disease progression in the GT 3 group and non-GT 3 group, after exclusion of patients with recurrence. (A)The cumulative incidence rates of OLDP; (B) The cumulative incidence rates of CLC; (C) The cumulative incidence rates of DLC; (D) The cumulative incidence rates of HCC. The level of significance was set at p<0.05 (Kaplan‒Meier estimates). Abbreviations: GT, genotype; OLDP, overall liver disease progression; CLC: Compensated liver cirrhosis; DLC: Decompensated liver cirrhosis; HCC, hepatocellular carcinoma.


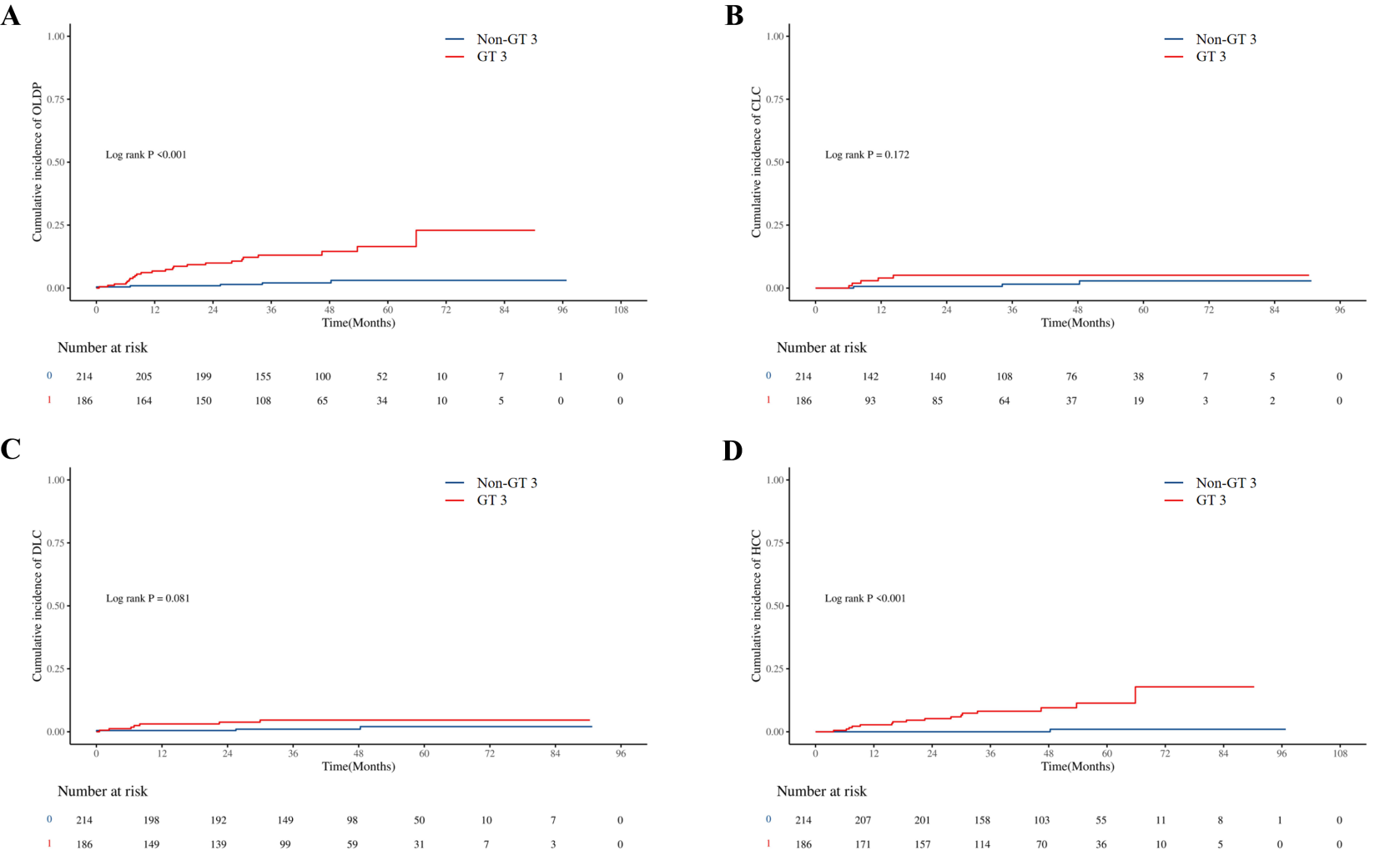

Supplement: Supplementary file 1 [file Table1.docx]
